# Microswimmers That Flex: Advancing Microswimmers with Templated Assembly and Responsive DNA Nanostructures

**DOI:** 10.1021/accountsmr.5c00009

**Published:** 2025-07-14

**Authors:** Taryn Imamura, Sarah Bergbreiter, Rebecca E. Taylor

**Affiliations:** † Department of Mechanical Engineering, 6612 Carnegie Mellon University, 5000 Forbes Avenue, Pittsburgh, Pennsylvania 15213, United States; ‡ Robotics Institute, Carnegie Mellon University, 5000 Forbes Avenue, Pittsburgh, Pennsylvania 15213, United States; ¶ Department of Biomedical Engineering, Carnegie Mellon University, 5000 Forbes Avenue, Pittsburgh, Pennsylvania 15213, United States; § Department of Electrical and Computer Engineering, Carnegie Mellon University, 5000 Forbes Avenue, Pittsburgh, Pennsylvania 15213, United States

## Abstract

The concept of micrometer-scale swimming robots, also known as microswimmers, navigating the human body to perform robotic tasks has captured the public imagination and inspired researchers through its numerous representations in popular media. This attention highlights the enormous interest in and potential of this technology for biomedical applications, such as cargo delivery, diagnostics, and minimally invasive surgery, as well as for applications in microfluidics and manufacturing. To achieve the collective behavior and control required for microswimmers to effectively perform such actions within complex, in vivo and microfluidic environments, they must meet a strict set of engineering criteria. These requirements include, but are not limited to, small size, structural monodispersity, flexibility, biocompatibility, and multifunctionality. Additionally, microswimmers must be able to adapt to delicate environments, such as human vasculature, while safely performing preprogrammed tasks in response to chemical and mechanical signals.

Naturally information-bearing biopolymers, such as peptides, RNA, and DNA, can provide programmability, multifunctionality, and nanometer-scale precision for manufactured structures. In particular, DNA is a useful engineering material because of its predictable and well-characterized material properties, as well as its biocompatibility. Moreover, recent advances in DNA nanotechnology have enabled unprecedented abilities to engineer DNA nanostructures with tunable mechanics and responsiveness at nano- and micrometer scales. Incorporating DNA nanostructures as subcomponents in microswimmer systems can grant these structures enhanced deformability, reconfigurability, and responsiveness to biochemical signals while maintaining their biocompatibility, providing a versatile pathway for building programmable, multifunctional micro- and nanoscale machines with robotic capabilities.

In this Account, we highlight our recent progress toward the experimental realization of responsive microswimmers made with compliant DNA components. We present a hybrid top-down, bottom-up fabrication method that combines templated assembly with structural DNA nanotechnology to address the manufacturing limitations of flexibly linked microswimmers. Using this method, we construct microswimmers with enhanced structural complexity and more controlled particle placement, spacing, and size, while maintaining the compliance of their DNA linkage. We also present a novel experimental platform that utilizes two-photon polymerization (TPP) to fabricate millimeter-scale swimmers (milliswimmers) with fully customizable shapes and integrated flexible linkers. This platform addresses limitations related to population-level heterogeneity in micrometer-scale systems, allowing us to isolate the effects of milliswimmer designs from variations in their physical dimensions. Using this platform, we interrogate established hydrodynamic models of microswimmer locomotion and explore how design and actuation parameters influence milliswimmer velocity. We next explore opportunities for enhancing microswimmer responsiveness, functionality, and physical intelligence through the inclusion of nucleic acid subcomponents. Finally, we highlight how our parallel research on xeno nucleic acids and interfacing DNA nanotechnology with living cells can enable the creation of fully organic, truly biocompatible microswimmers with enhanced functionality, improving the viability of microswimmers for applications in healthcare, manufacturing, and synthetic biology.

## Graphical abstract

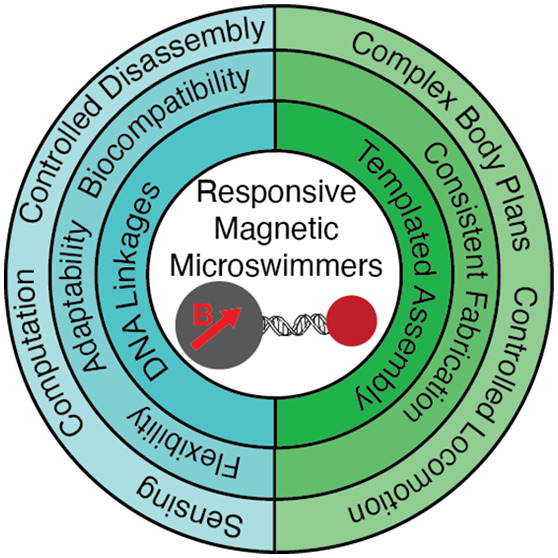

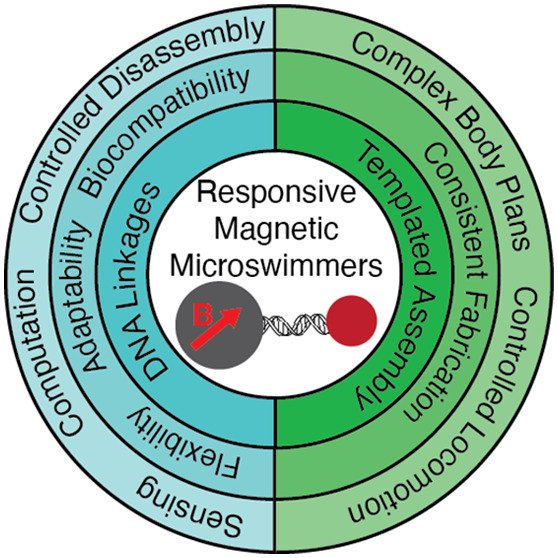

## Introduction

In the 1966 film *Fantastic Voyage*, a group of scientists are shrunk to the size of microbes and, in their microscopic submarine, traverse the vasculature of a scientist to locate and dispel a blood clot. This film has been extensively adapted in media such as *The Magic School Bus* and *Big Hero 6*, and continues to inspire scientists as they develop microscopic robots for medical therapies.^1^ Although it is a work of science fiction, this film illuminates key requirements that micrometer-scale swimming robots, or “microswimmers”, must meet to safely operate in the human body. To become practical for manufacturing and biomedical applications, such as targeted cargo delivery, diagnostics, and minimally invasive surgery, microswimmers must be able to navigate in and adapt to intricate environments, such as the human vasculature.^1–4^ Additionally, microswimmer populations must be structurally identical (i.e., monodisperse) to act collectively and precisely perform tasks in response to specific signals.^5,6^ Finally, microswimmers must be biocompatible and capable of accomplishing tasks without triggering errant immune, toxicological, or environmental responses.^3,4^

These requirements generate stringent design metrics that microswimmers must fulfill to become viable for healthcare, manufacturing, and synthetic biology applications (Figure 1). For instance, individual microswimmers must be small, ideally nanometers to tens of micrometers in scale, to locomote in constricted parts of the human body such as capillaries, which have diameters of 5 to 10 μm and are our smallest vessels. Microswimmers must also be flexible and biocompatible enough to navigate through these systems without interfering with or damaging delicate tissues. They must also be multifunctional and able to reconfigure their morphology or perform specific tasks in response to physiological triggers. Finally, fabrication methods must be capable of producing large, monodisperse populations of microswimmers with a variety of body plans.

**Figure 1:**
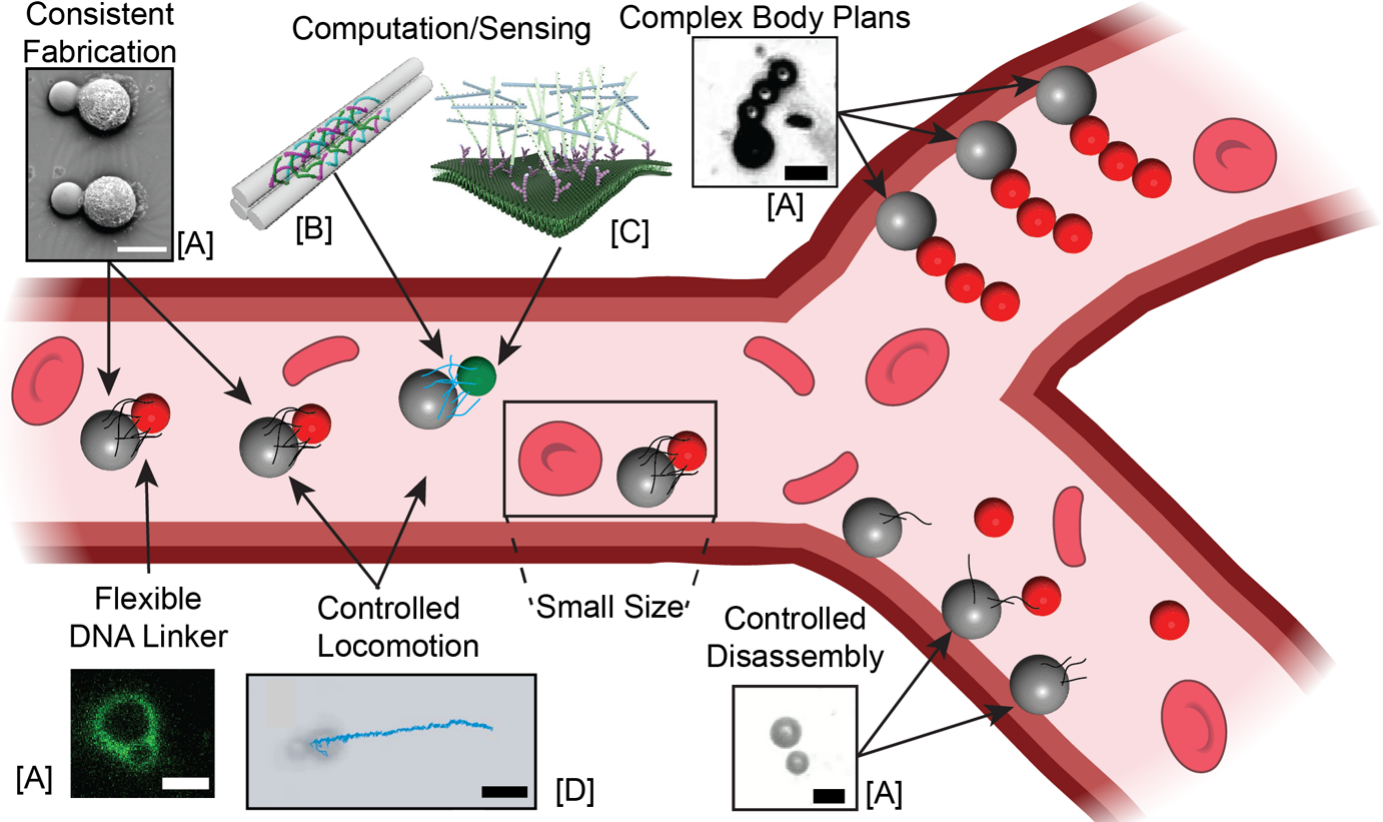
Schematic of the criteria that microswimmers must meet for safe and practical deployment in the human body. Inset scale bars: 10 μm. Images labeled [A] reproduced with permission from ref 7. Copyright 2025 The Authors. Images labeled [B] reproduced with permission from ref 8. Copyright 2020 The Authors. Images labeled [C] reproduced with permission from ref 9. Copyright 2023 The Authors. Images labeled [D] reproduced with permission from ref 10. Copyright 2020 The Authors. Red blood cell illustrations were from NIAID NIH BIOART Source (bioart.niaid.nih.gov/bioart/14).

Biological organisms already meet many of these requirements, and since the release of *Fantastic Voyage*, researchers have leveraged biological components to create biohybrid microswimmers with improved functionality. By incorporating materials like living cells, bacteria, proteins, and DNA as subcomponents in robotic systems, researchers have created microswimmers that can perform robotic tasks, such as targeted cargo delivery, toxin detection, and minimally invasive surgery.^1,4,11,12^ Naturally information-bearing biopolymers, such as peptides, DNA, and RNA, can provide programmability, multifunctionality, and chemical reactivity, making them powerful components for giving microswimmer systems reconfiguration and responsive capabilities while maintaining their biocompatibility.^13,14^ Like the premise of *Fantastic Voyage*, nanostructured micro- and mesosystems formed through bottom-up assembly once sounded like science fiction, but recent advances in nucleic acid nanotechnology have enabled unprecedented abilities to engineer functional systems at the nanometer and micrometer scales, making the self-assembly of micron-scale structures a reality.^12,15,16^ DNA is a useful engineering material because it has predictable and well-characterized material properties, allowing us to overcome challenges associated with modular microstructure assembly.^15^ DNA-architected materials can also organize molecular and nanoscale components with nanometer-scale resolution, often reliably and with high throughput, unlocking the potential for the fabrication of multimaterial microscale 3D electronics, dynamic and responsive metamaterials, and even synthetic biological machines.^17^ Moreover, DNA is compatible with other biopolymers such as peptide nucleic acid (PNA), peptides, and proteins, giving researchers a platform for combining structures such as protein vaults, phase-separating condensates, and even living cells to create multifunctional, biocompatible robotic systems.^8,18,19^

In this Account, we highlight our recent progress toward the experimental realization of microswimmers made with compliant DNA components. By combining templated assembly with structural DNA nanotechnology, we leverage the 2D control and submicron assembly precision of these two methods to construct microswimmers with flexibility, responsiveness, and enhanced structural complexity. Using a novel millimeter-scale experimental platform, we interrogate velocity predictions from hydrodynamic microswimmer models. We then demonstrate how including DNA nanostructures as subcomponents in microswimmer systems can enhance their responsiveness and outline opportunities for leveraging these structures to expand microswimmer functionality. Finally, we highlight how our parallel research on xeno nucleic acids (XNAs) and cell-interfacing DNA nanotechnology can enable the future creation of fully organic, specialized microswimmers. Looking forward, these advancements will enhance the viability of microswimmers for applications in biomedicine, synthetic biology, and manufacturing.

## Templated Assembly of Flexible Microswimmers

Microswimmers constructed using soft materials, such as information-bearing biopolymers, can gain functional advantages like deformability, biocompatibility, and reconfigurability,^2–4,20^ allowing them to navigate intricate spaces like the human vasculature.^2,6^ Moreover, enhanced flexibility can improve swimming velocity for articulated microswimmers.^11,21^ Larger-scale soft microrobots (from hundreds of micrometers to several millimeters) have successfully performed tasks such as cargo delivery, navigation, and adaptive locomotion.^2,3^ However, miniaturizing soft microswimmers for smaller-scale applications (from the nanoscale to tens of micrometers) presents manufacturing challenges.

As they become smaller, the influence of low Reynolds number hydrodynamics on microswimmer locomotion becomes greater.^22,23^ In these environments, viscous forces dominate, and characteristics such as size, symmetry, and geometry govern how quickly microswimmers locomote.^22,24^ Consequently, as these systems are scaled down, structural polydispersity leads to variability in swimming speed and direction, hindering motion control and path planning.^21,25^

Methods such as lithography and microfluidic processes have been used to construct populations of rigid microswimmers with submicron precision;^1,2^ however, these techniques may respectively limit the reconfigurability and biocompatibility of these systems. Colloidal microswimmers have been assembled via external fields, but these structures are often highly heterogeneous across populations.^26^ Although physical templates can guide colloidal particles into precise 2D configurations, microswimmers produced with this method are typically rigid, limiting their adaptability and reconfigurability.^2,27^ Consequently, fabrication methods capable of constructing microswimmers with flexible components and submicrometer precision are needed. Previous research has used DNA to construct flexible DNA-microparticle actuators, such as flagellar components, which were capable of locomotion.^11,25,28,29^ For a more detailed discussion of existing DNA microswimmers and magnetically actuated microsystems, refer to the Supporting Information (Section S1). Building on this work, our team developed a hybrid top-down, bottom-up fabrication approach that integrates templated assembly by selective removal (TASR) with single-stranded tile (SST) DNA nanotubes to construct articulated microswimmers with flexible linkages and controlled body designs (Figure 2A).^10^

**Figure 2:**
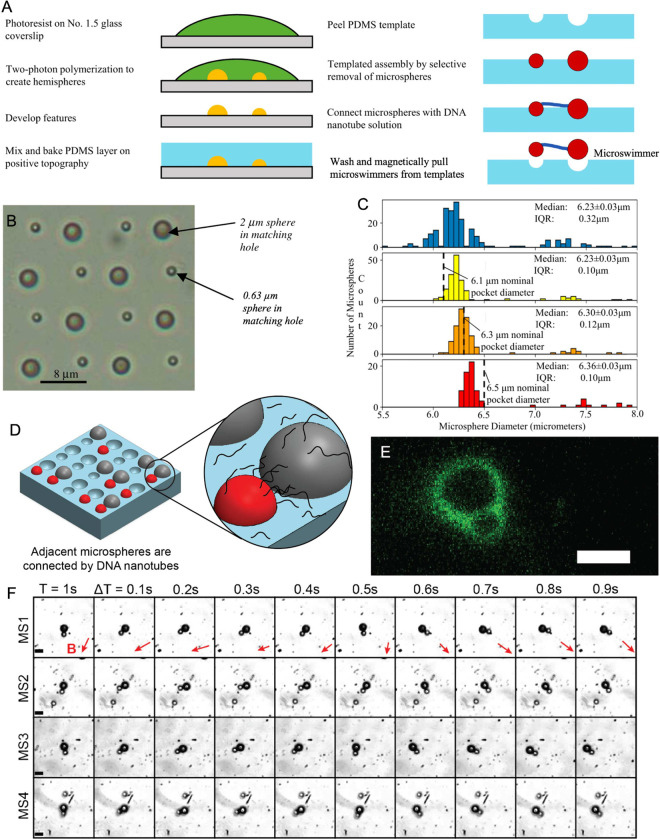
Construction of colloidal microswimmers with flexible DNA linkages and controlled particle placement and size. (A) Schematic showing the fabrication of templates and the assembly of polymer and DNA components into flexibly linked microswimmers. Adapted with permission from ref 10. Copyright 2020 The Authors. (B) Image of microspheres assembled in a TASR template demonstrating size selectivity. Reproduced with permission from ref 30. Copyright 2008 IOP Publishing. (C) Histograms showing diameters of input microsphere population (blue) and diameters of microspheres retained in pockets with nominal diameters of 6.1 μm (yellow), 6.3 μm (orange), and 6.5 μm (red), respectively. Reproduced with permission from ref 10. Copyright 2020 The Authors. (D) Schematic illustrating the linkage of assembled microspheres via DNA nanotubes. (E) Fluorescence microscopy image confirming conjugation of the DNA nanotube linkage to microspheres. (F) Images from captured video of microswimmers made using this method actuated by magnetic fields. Magnetic field directions are shown by red vectors at the top of each column. Scale bars in panels E and F are 10 μm. Reproduced with permission from ref 7. Copyright 2025 The Authors.

TASR is a template-based sorting method that enhances control over particle size and 2D placement, reducing the dimensional variability of assembled synthetic and biological components (Figure 2B).^30,31^ TASR is also compatible with a range of microparticle geometries and materials, making it an attractive method for constructing organic and biohybrid microsystems from diverse components. We demonstrated that tuning the diameter of TASR template pockets enhances control over particle size (Figure 2C), facilitating parallel selection of homogeneous microsphere subpopulations from heterogeneous inputs and supporting the controlled assembly of customizable microswimmers from diverse components (Figure 2D).

We utilized the filtering and placement capabilities of TASR to create a minimal yet functional microswimmer design that showcased the geometric precision afforded by this method: a large, ferromagnetic microsphere attached to a smaller, nonmagnetic microsphere at a designated distance by compliant 10-helix, biotinylated DNA nanotubes (Figure 2D,E).^10^ These nanotubes are formed using the single-stranded tile (SST) approach, which forms length-distributed, micron-scale linkages. The Supporting Information provides additional information on SST nanotube structure (Section S2 and Figure S1). This hybrid method produced articulated microswimmers that were responsive to magnetic fields (Figure 2F) and possessed the flexible linkages needed to break the “Scallop Theorem,” which states that microswimmers must undergo nonreciprocal motion to achieve net propulsion in low Reynolds number environments.^10,22,23^ While we chose SST DNA nanotubes as our linkage because of their micrometer-scale length, tunable stiffness, and precise diameter control,^32^ other biopolymers like RNA and PNA could also be used depending on the desired application.^8,33^

To enable their real-world use, microswimmers must be capable of operating in unstructured environments. However, studying microswimmers under nonidealized conditions (i.e., suspended in a liquid and subjected to external stimuli such as flow) presents significant challenges related to isolating intentional microswimmer locomotion and conducting path planning. To address this challenge, our team recently developed a technique that uses tracked position data from magnetic and nonmagnetic fiducial microspheres to capture the effects of fluid flow and magnetic field gradients on colloidal microswimmer locomotion.^34^ Using this approach, we determined that, when actuated by a 1 Hz oscillating magnetic field, our microswimmers locomoted at velocities ranging from 0.4 to 0.9 μm s^–1^ (0.02–0.05 BL s^–1^), indicating that they are not rigidly linked systems.^10,34^ Although applied magnetic fields can control microswimmer orientation, we observed variation in their swimming directions, suggesting differences in their internal magnetic orientations. Swimming speeds were also variable, likely due to polydispersity in both the length and number of DNA nanotubes connecting each microswimmer, which affect linkage stiffness and mechanics. To achieve coordinated swarm behavior in future studies, variation in microswimmer magnetization and linkage mechanics must be addressed.

By integrating lithography, templated assembly, and DNA nanotechnology, our hybrid method preserves micrometer-scale control over particle spacing, size, and arrangement while achieving nanometer-scale feature resolution, enabling the precise assembly of flexibly linked microswimmers. Looking forward, this approach can be used to design application-specific microswimmers and scaled for assembling populations of structurally uniform colloidal robots.

## Microswimmers with Flexible, Complex Body Plans

Compared to individual microswimmers, swarms are often better equipped to perform robotic tasks.^5,6^ Drawing inspiration from biological systems, researchers have created swarms of micro- and nanorobots with behaviors such as self-organization, adaptability, and robustness, allowing them to function with greater efficiency and resilience.^5^ Enhancing the structural complexity (i.e., the number and arrangement of components) of individual microswimmers can further improve the versatility of these systems. However, constructing populations of microswimmers with flexible components while maintaining their functionality and structural complexity is challenging, particularly for applications that require high-throughput, reproducible fabrication of nano- and microrobotic systems.^3,4^ While top-down methods like optical, opto-thermophoretic, and plasmonic tweezers provide precise positioning of individual particles,^26,35^ their low throughput limits scalability (Figure S2A,B). On the other hand, modular microswimmers have been assembled using external stimuli such as electric, magnetic, acoustic, or light-based fields, but the resulting structures are often highly heterogeneous (Figure S2C,D).^11,26^

While physical templates offer greater precision and enable the assembly of microscale components into a range of arbitrary 2D designs (Figure S2E),^27^ these methods typically produce inflexible microswimmers or suffer from low assembly yields. These limitations reduce the agility, functionality, and complexity of these systems (Figure S2F), highlighting the need for assembly methods that can construct microswimmers with complex body designs and flexible linkages.^2,27^

Building on our previous work,^10,36^ we demonstrated that tailoring template material to desired components significantly enhances TASR-based assembly, enabling the fabrication of colloidal microswimmers with more components.^7^ TASR assembly is influenced by the strength of surface interactions, and as a result, modifying template or component surface energies will directly impact component assembly.^30,31^ High-surface-energy materials are more adhesive to each other.^37^ We therefore used recent advances in polycarbonate heat (PCH) molding to explore the use of polycarbonate, a high surface energy templating material, to improve pocketing yields (Figure 3A).^7,36,38^

**Figure 3:**
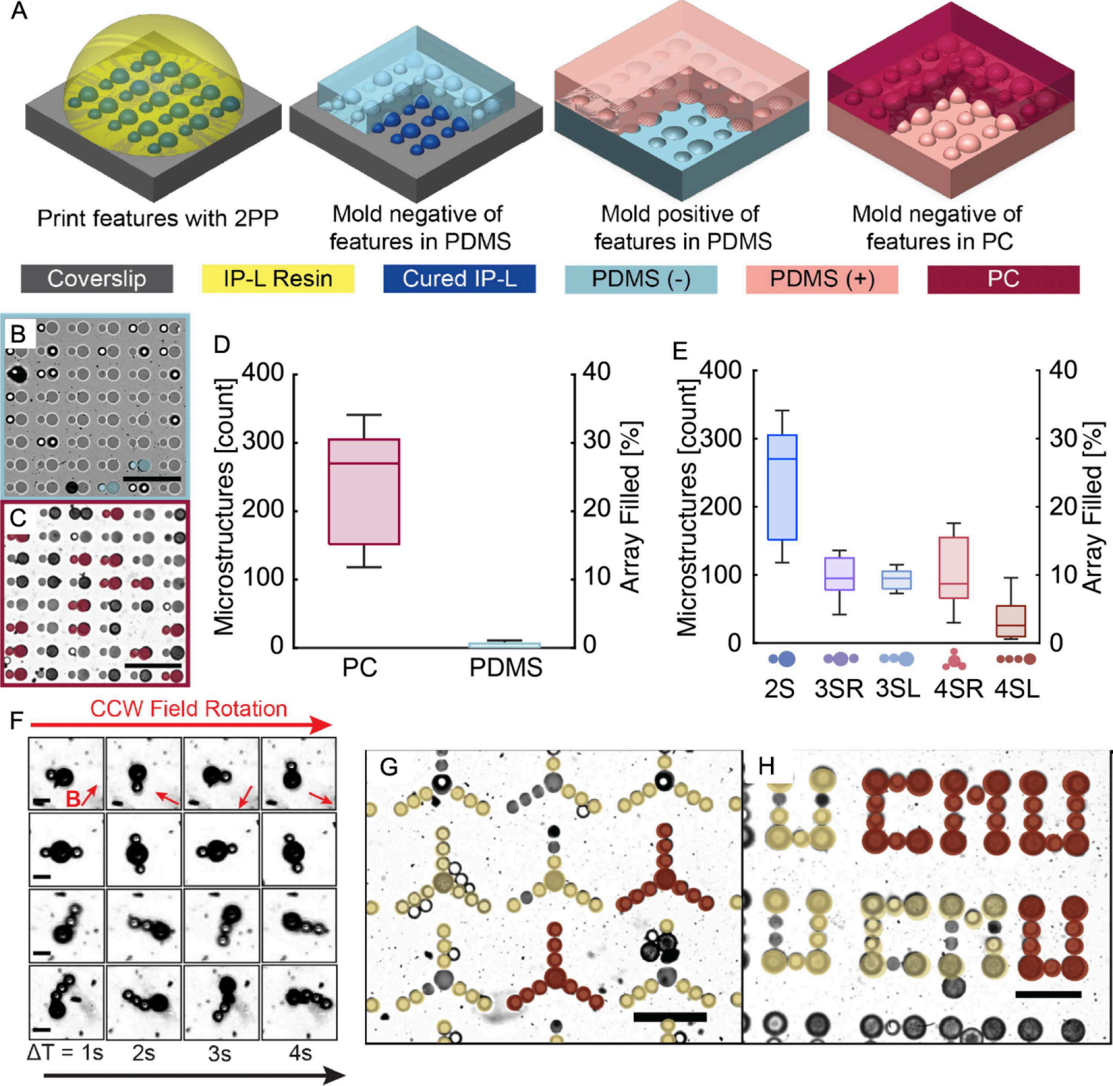
(A) Schematic showing the fabrication of polycarbonate (PC) and polydimethylsiloxane (PDMS) TASR templates made using our adapted PCH molding process. (B, C) Optical micrographs showing the templated assembly of polystyrene microspheres in the PDMS (blue) and PC (pink) templates. Assembled microspheres are identified by color overlays. Scale bars are 40 μm. (D) Comparison of the assembly yields for two-sphere microswimmers in the PDMS and PC templates across 5 replicates. (E) Comparison of pocketing yields for radial and linear microswimmer designs requiring two, three, and four microspheres. (G) Images of microswimmers with enhanced complexity constructed using PC templates. Microswimmers are suspended in solution and actuated by rotating magnetic fields. Magnetic field vectors are represented by red arrows in each frame. Scale bars are 10 μm. (G, H) Higher complexity 10-sphere microrotors and 26-sphere “CMU” microstructures were assembled using the PC template. Red overlays represent complete structures, and yellow overlays denote partially assembled structures. Scale bars are 40 μm. Figures reproduced with permission from ref 7. Copyright 2025 The Authors.

Tuning template surface energy to our desired components through material choice generated assembly improvements of more than 100-fold for two-sphere microstructures and facilitated higher-throughput production of simple two-sphere microswimmers (Figure 3B–D). These substantial assembly improvements also allowed us to investigate how microswimmer design complexity, specifically the number of microspheres and patterning of micropockets, impacts self-assembly rates. We found that assembly yields declined as the number of microspheres required to complete an assembly increased but that micropocket arrangement did not have an observable impact on microstructure assembly (Figure 3E).

Building on these improvements, we successfully constructed DNA-linked microswimmers with enhanced complexity and controlled particle placement (Figure 3F). Further evidence that the microswimmers are flexibly linked is provided in Supporting Video S6 of Imamura et al.^7^ Using these assembly enhancements, we also demonstrated precise templating of microstructures that would be challenging to achieve with other templated methods, such as 10-sphere microrotors and a 26-sphere “CMU” pattern (Figure 3G,H).

This study addresses a critical gap in the colloidal microswimmer literature by introducing a scalable process for manufacturing microswimmers with controlled geometries and compliant linkages. By overcoming prior challenges in controlling microparticle spacing, patterning, and size, this approach expands the design space for articulated microswimmers. It also enables higher-throughput microswimmer fabrication, laying the groundwork for future swarm-based applications. While variable assembly yields and limited recovery from templates pose challenges for scaling up microswimmer production, these issues can be mitigated by using multiple templates in parallel and developing more effective recovery techniques.^7^ While this method offers greater versatility in microswimmer design and increased fabrication throughput, tighter regulation of microparticle magnetization, DNA linkage length, and DNA linkage conjugation are needed to achieve monodisperse swimming speed and direction, which will be required for swarm studies. However, our innovations now allow us to interrogate how design characteristics, such as microsphere arrangement, particle type, and linkage flexibility, impact microswimmer locomotion.

## Model-Based Design Guidelines for Flexible Milliswimmers

So far, our work has improved the structural precision and complexity of colloidal microrobots by combining physical templates with compliant DNA linkages.^7,10,36^ However, these colloidal systems still displayed polydisperse swimming behavior, which may result from variations in common microscale building materials, such as microspheres.^10^ In low Reynolds number environments, this variation alters physical attributes of the microswimmers, such as their link shape, size, and aspect ratio, and impacts their swimming behavior.^3,22^ For this technology to become practical for in vivo applications, researchers must understand how these design parameters impact microswimmer locomotion. Previous studies have modeled articulated microswimmers using planar, hydrodynamic drag models to examine this design space.^23,24^ However, while experimental validation of these models is needed, structural polydispersity in colloidal microswimmer populations hinders our ability to accurately assess these model predictions.^25^

To address these limitations and isolate the effects of microswimmer designs from variations in their physical dimensions, our team developed an experimental platform that leverages two-photon polymerization (TPP) to construct millimeter-scale swimmers with fully customizable shapes and integrated flexible linkers.^21^ Using this platform, we built and tested a series of articulated “milliswimmers” (2.21 mm total length) with two link geometries: cylindrical, which allowed us to interrogate velocity predictions from the first- and second-order models, and spherical, which matched the shape of our colloidal systems (Figure 4A,B).^21^ These designs also included internal cavities that provided our milliswimmers with buoyancy control. We observed improved structural monodispersity for the printed milliswimmers, affording us enhanced control of swimming direction and repeatability of milliswimmer performance compared to our micron-scale system. Although these milliswimmers were orders of magnitude larger than the colloidal microswimmers discussed in previous sections, we maintained a dynamic similarity between these two systems by conducting all millimeter-scale experiments in Percoll. When deployed in this viscous fluid, the Reynolds number of the milliswimmers was ∼1 × 10^–4^, while that of the colloidal microswimmers was ∼1 × 10^–5^.^21^ Both values are well below one, indicating that inertial effects should be negligible in both systems. Because microswimmers must have tunable, predictable swimming behavior to be used in commercial and medical applications, we chose swimming velocity as our comparison metric for exploring the design and control of various two-link systems.

**Figure 4:**
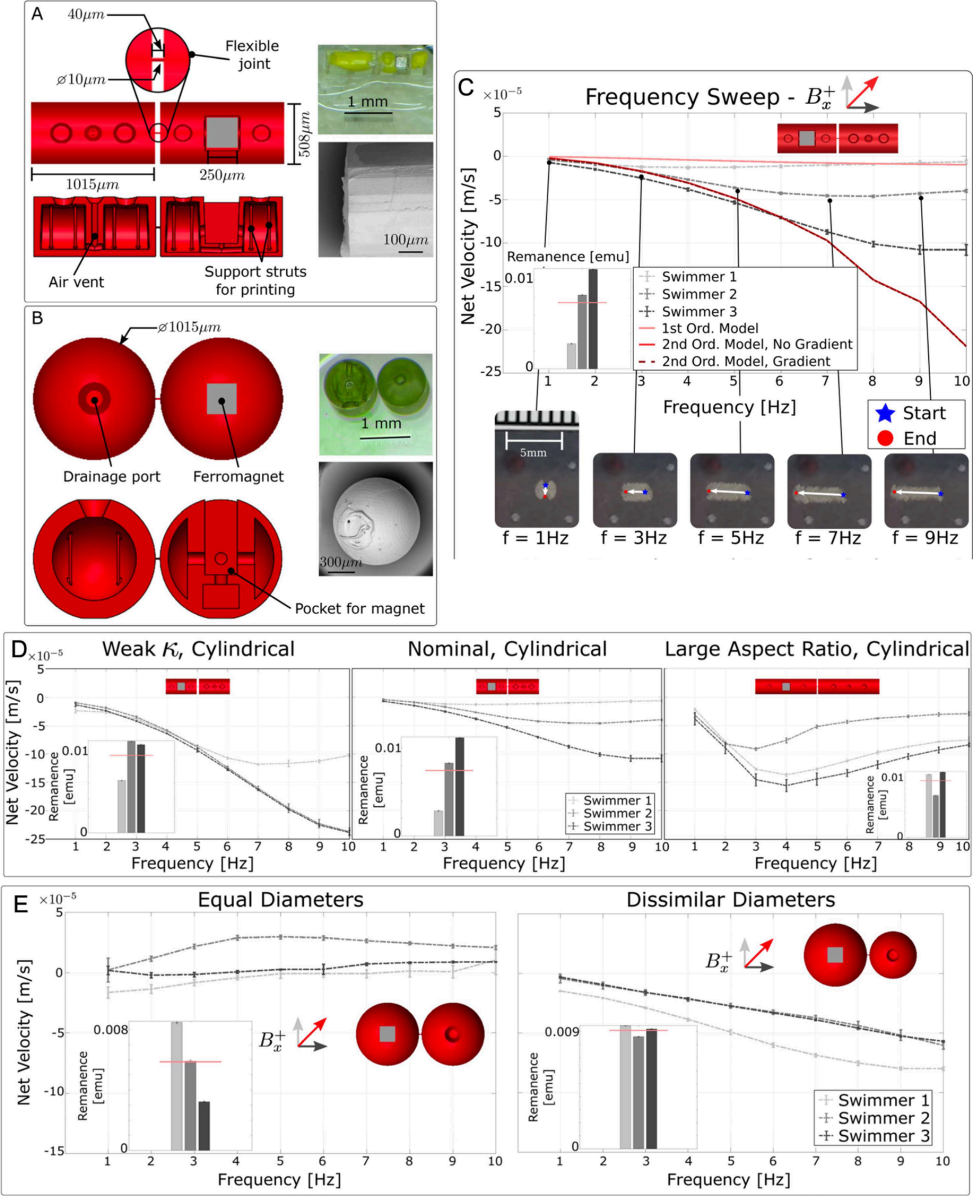
Millimeter-scale swimmers printed with two-photon polymerization controlled for manufacturing variance. CAD designs for (A) cylindrical and (B) spherical milliswimmers are shown in red. Interior chambers provide buoyancy during experimentation, and support struts provide proper structure formation during printing. Drainage ports allow excess photoresist to flow out after printing, and a ferromagnet provides actuation in magnetic fields. The flexible joint simulates the DNA nanotube linkage and is 10 μm in diameter and 40 μm in length for both nominal designs. The top right insets of (A) and (B) show images of the completed milliswimmer, and the bottom right insets show the detailed surface finish of each. (C) Comparison of first- and second-order models compared to frequency sweep data for nominal, cylindrical milliswimmers. Time-lapse images indicate milliswimmer motion at the indicated frequencies. (D) Trends in milliswimmer net velocity due to variation in the torsion spring constant (left) and link aspect ratio (right) were observed. Data from the nominal milliswimmer design is provided for comparison (center). (E) Net velocity for spherical milliswimmers with equally sized links (left) and dissimilarly sized links (right) was also assessed. Inset bar plots show the magnetic remanence of each milliswimmer with the mean plotted as the red line. Figures reproduced with permission from ref 21. Copyright 2023 The Royal Society of Chemistry.

To test our milliswimmer platform, we developed a first-order hydrodynamic drag model. Like common models in the literature, our model is planar, considers inertial effects to be negligible, and represents our milliswimmers as a three-link system consisting of two cylinders connected by a torsional spring (Figure S3A,B).^23,39^ We then actuated milliswimmers with oscillating magnetic fields and recorded how their net velocities changed with respect to field frequency, link aspect ratio, joint stiffness, and link shape, comparing these experimental results to net velocity predictions from our quasistatic model. These studies revealed that purely quasistatic models do not accurately capture key features of milliswimmer locomotion (Figure 4C).^21^ Specifically, these models underestimate milliswimmer net velocities and predict a monotonic velocity increase with driving frequency. In contrast, our experimental results show that velocities plateaus at higher frequencies. To capture this behavior, we recast our model as a second-order system that includes inertial effects. In this model, the analytically predicted hydrodynamic drag coefficient (*k*_*t*_) overestimated milliswimmer velocities, so we fit the value to our experimental data. While incorporating inertia improved the accuracy of our velocity predictions, neither model captured velocity plateaus at higher frequencies (Figure 4C),^21^ suggesting that a key factor affecting dynamic similarity may still be missing from current models. While our original Reynolds number calculations used net velocity, recalculating them using the instantaneous velocity of each link revealed much higher values (0.1 < Re < 1.2), indicating greater inertial effects than initially assumed.

The relative monodispersity of our printed milliswimmer system illuminated design and actuation trends that can be used to optimize swimming velocity (Figure 4D). In cylindrical milliswimmers, actuation frequencies could be adjusted to attain faster swimming velocities, and changing link aspect ratios shifted the frequency at which peak swimming velocity occurred.^21^ In particular, milliswimmers with larger link aspect ratios swam faster at lower actuation frequencies. Increasing milliswimmer linkage flexibility also resulted in faster swimming, highlighting the importance of linkage flexibility.^21^

To evaluate a system more analogous to colloidal microswimmers, we also developed first- and second-order models for spherical milliswimmers (Figure S3C,D). Both models predicted that spherical milliswimmers, regardless of their design, would not achieve net translation. However, experimental results revealed that milliswimmers with dissimilar link diameters attained net displacement while milliswimmers with links of the same diameter did not (Figure 4E), a result that aligns with previous colloidal microswimmer results.^10,34^ While previous studies have primarily represented microswimmer links as cylinders, our results highlight an opportunity for developing hydrodynamic models that more faithfully capture the spherical geometries of colloidal systems. For detailed model derivations, we refer readers to the Supporting Information of ref 21.

Looking forward, our models provide an improved understanding of how key parameters, such as magnetic moment, link geometry, torsional spring stiffness, and fluid viscosity, may influence articulated milliswimmer locomotion. These results also highlight a need within the field to investigate appropriate metrics of dynamic similarity for microswimmer systems and, more broadly, to assess how well dynamic models align with their experimental counterparts and develop models that incorporate fluid-structure interactions, system compliance, and deformation.

## Enhancing Microswimmer Responsiveness with DNA Nanostructures

The integration of onboard sensors and soft materials can give biohybrid micro- and nanoswimmers the ability to respond to environmental stimuli, such as mechanical forces, chemical signals, and changes in temperature or pH.^4,6,40^ Moreover, autonomously changing morphology and locomotion mechanisms in response to environmental signals can allow microrobots to better navigate confined biological spaces and selectively release cargo, enhancing their biomedical potential.^2,6^ While larger robots have achieved autonomous reconfiguration in response to discrete signals, smaller-scale microswimmers often lack the ability to autonomously sense and respond to changes in their environment and require a human or a computer in the loop to accomplish complex tasks.^2,6,40^ Additionally, reconfigurable microswimmers must be carefully designed to repeatably and predictably respond to desired signals without triggering unwanted interactions with other physiological systems, such as the immune system.^2,4,6^

Given the predictable behavior and biocompatibility of DNA, and the tunable mechanics and addressability of DNA nanotechnology, DNA is an advantageous nanomaterial for constructing nanoscale systems.^15^ Moreover, these structures can be programmed with responses that are tailored to particular stimuli, decreasing cross-talk between signal inputs and outputs and minimizing the potential for errant immune, toxicological, or environmental responses.^14^ DNA nanostructures can also be tuned to respond to particular signal thresholds,^14,19^ making DNA an attractive biopolymer for building customizable, responsive microswimmer systems at small scales.

To illustrate how the inclusion of DNA nanostructures can make microswimmers responsive to biochemical signals, we demonstrated the nuclease-triggered disassembly of a DNA-linked colloidal microswimmer.^7^ We exposed one of our microswimmers to deoxyribonuclease I (DNase I), an endonuclease enzyme that nonspecifically cleaves double-stranded DNA (dsDNA) by severing the phosphodiester bonds that comprise the DNA backbone (Figure 5A).^41^ Before exposure to DNase I, the microswimmer was actuated by oscillating magnetic fields to verify that DNA nanotubes formed a robust linkage between the microspheres (Figure 5B). After incubating with DNase I for 50 min and 52 s (3052 s), we observed the microspheres separating and reconnecting multiple times before the microswimmer finally disassembled (Figure 5C).^7^

**Figure 5:**
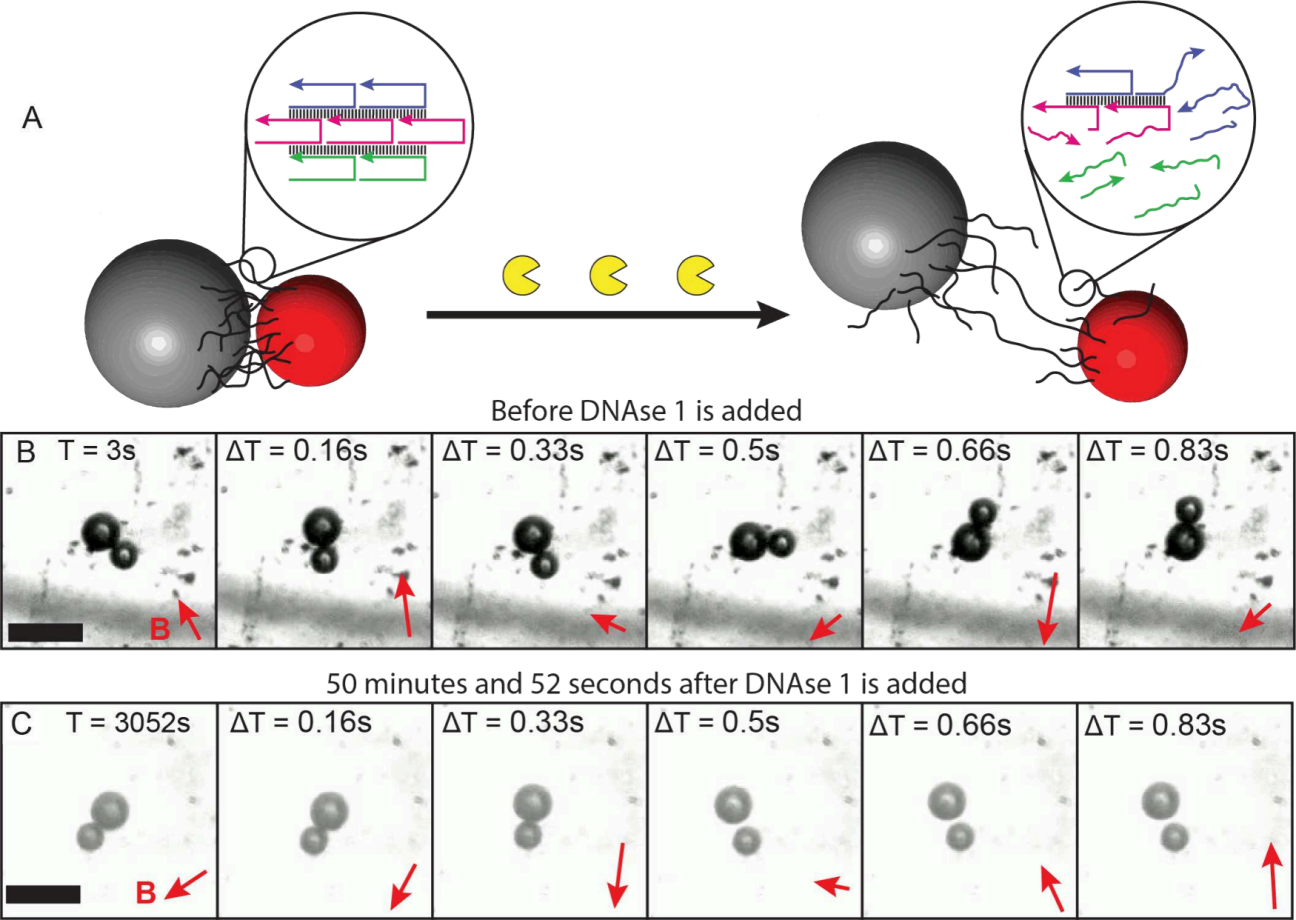
Real-time microswimmer reconfiguration enabled by DNA. (A) A diagram illustrating how DNase I cleaves the DNA linkage of the microswimmer. (B) Before exposure to DNase I, the microswimmer is responsive to magnetic fields, implying robust DNA nanotube connections. (C) After incubating with DNase I for 50 min and 52 s (*T* = 3052 s), the same microswimmer decouples and is no longer responsive to magnetic fields. Scale bars are 20 μm. The magnetic field directions are shown by red vectors, and the time steps are shown in each image. Reproduced with permission from ref 7. Copyright 2025 The Authors.

The deconstruction of this microswimmer is a conceptual demonstration that highlights future opportunities for expanding microswimmer functionality through the incorporation of responsive subcomponents. By leveraging the material properties of individual structures such as DNA nanotubes, these robots can gain embodied intelligence. In this example, the DNA linkage allowed the microswimmer to respond to a chemical signal in its environment—the presence of DNase I—by autonomously altering its morphology.^7^

Following this reconfiguration, the microswimmer was not responsive to magnetic fields and was incapable of net propulsion, suggesting that DNA properties can be leveraged to autonomously toggle microswimmer motility. Furthermore, the detachment of the nonmagnetic bead, which could be viewed as a cargo, after exposure to the enzyme highlights the potential of DNA-mediated responsiveness for targeted cargo delivery.

In this proof-of-concept study, DNase I was used to trigger microswimmer disassembly, highlighting both a potential vulnerability of DNA-based systems in physiological environments and an opportunity for controlled degradation in targeted delivery. However, due to low salt denaturation and enzymatic degradation, various strategies may be needed to stabilize DNA nanostructures for biological applications. As described in Stephanopoulos et al., approaches such as covalent cross-linking and encapsulation by polymers or lipid bilayers can enhance DNA nanostructure stability in physiological conditions.^42^ Alternatively, nucleic acid mimics, such as XNAs, or chemically modified nucleotides also provide exciting opportunities for improving microswimmer biostability while retaining the dynamic responsiveness of their DNA components.^42,43^ This experiment was the first demonstration of using functional DNA nanostructures to create responsive multimaterial microswimmers. With further development, these nanostructures can act as onboard sensors, enabling autonomous behaviors such as cargo delivery, signal tracking, self-assembly, and obstacle avoidance.

## Xeno Nucleic Acids

For microswimmers to become practical for biomedical and manufacturing applications, control over their response to signals (i.e., when and how they disassemble or release cargo) is essential. However, maintaining this control in nonlaboratory settings is challenging. For example, physiological environments pose risks to DNA-containing microrobots, and therefore mitigations may be necessary to avoid low-salt denaturation and enzymatic degradation of DNA nanostructures in physiological media. In general, three strategies have been employed to enhance the stability of DNA nanostructures: (1) altering the buffer conditions or nanostructure designs, (2) altering external architecture via cross-linking, and (3) encapsulating the nanostructures.^42^

Our group is pursuing a fourth option for stabilizing nucleic acid-based microswimmers in physiological environments: the inclusion of nucleic acid mimics. Also known as xeno nucleic acids (XNAs), these structures comprise a group of synthetic genetic polymers with modifications to their sugars, backbones, and bases.^43^ Many of these polymers have emerging applications in biointerfacing nanotechnologies and antisense therapeutics due to their enhanced binding affinities, immune recognition, and enzymatic resistance. Two such examples are locked nucleic acids (LNA) and peptide nucleic acid (PNA). In the LNA backbone, a methylene bridge between the 2′-oxygen and 4′-carbon constrains the ribose ring and increases the binding affinity of LNA.^44,45^ The uncharged N-(2-aminoethyl)­glycine backbone of PNA allows for high-affinity binding and remarkable strand invasion properties, but that same lack of charge can lead to solubility issues.^46^ Additionally, α-l-threose nucleic acid (TNA), whose backbone contains a four-carbon threose instead of a natural five-carbon ribose, has demonstrated emerging applications in numerous nanotechnological areas, such as molecular computing and synthetic biology.^47^ Chimeras made of DNA and TNA also have the potential to protect DNA origami structures against exonuclease I and DNase I.^48^

Our team has explored the use of peptide nucleic acid (PNA), in particular gamma-modified γPNA, for the formation of micron-scale nanofibers.^8,49^ The incorporation of backbone gamma modifications and charged peptides can enhance the solubility of PNA, and we have demonstrated that PNA nanofibers can be formed in aprotic organic solvent mixtures that would typically destabilize DNA nanostructures. We have further developed isothermal formation processes to enable the rapid and scalable formation of these structures.^50^ Like LNA and TNA, PNA can bind strongly to DNA, RNA, and itself, illustrating the potential for PNA nanotechnologies to stabilize nucleic acid nanotechnology in environments that would typically degrade natural nucleic acids. The following review provides further discussion of emerging 3-dimensional XNA nanotechnologies.^51^ In the future, these advances in XNAs will facilitate the construction of nucleic acid microswimmers that are stable under a range of physiological conditions, broadening the environments in which robots can be deployed.

## Opportunities for Microparticle Composition

In our DNA microswimmer platform, the nonmagnetic microparticle is essentially a cargo that can be dropped on demand, as shown in Figure 5. The selection of cargo type could transform our DNA microswimmer into a range of specialized microrobots. If one of the polymer microspheres were replaced with a living cell, this system could become a xenobot. To enable this capability, following advances by Akbari et al. in engineering cell function with DNA origami,^52^ our team has demonstrated anisotropy-based technologies to interface with the plasma membrane as well as the glycocalyx of cells (Figure S4A–C).^53,54^ We have shown that nanoparticle shape and valency, as well as placement of decorations, can improve binding and even enable conditional specificity. Likewise, these factors can modulate both intracellular delivery and nuclear uptake (Figure S4D).^18^

Building off of advances reported by Ohmann et al. for reducing cholesterol-driven aggregation of DNA nanostructures (Figure S4E),^55^ we have also applied and validated this nonaggregating approach for one-step labeling of cells using our cholesterol-decorated anisotropic DNA nanoparticles.^18^ Furthermore, we have shown that our approach is mechanically delicate enough to enable nonactivating attachment to highly mechanosensitive human blood platelets.^56^

Should the mechanical stimulation from microswimmer drag be an issue, we have also developed a DNA-based “nanoshell” encapsulation technique that enwraps living cells with a structured assembly of cross-linked DNA nanorods (Figure S4F).^9^ This process effectively creates a temporary external cytoskeleton that improves the survival of encapsulated cells exposed to mechanical stimulation from osmotic swelling, centrifugation, and syringe injection. Together, these technologies highlight the potential of DNA microswimmers in both cell therapeutics and cellular assembly applications.

Looking forward, these approaches could likewise enable the cargo-carrying of synthetic cells, vesicles, and even molecular condensates, thus establishing DNA microswimmers as potential transport platforms for applications in synthetic biology and biomanufacturing. Finally, while it is still difficult to create nucleic acid structures with high yield and fixed sizes at the micrometer scale, recent advances indicate that all-DNA microswimmers may soon be a possibility. For example, Gigadalton-scale DNA origami assemblies have been successfully assembly with increasingly high yields and capabilities like virus encapsulation (Figure S5A,B).^57,58^ The assembly of even larger DNA-based, micron-scale structures has been demonstrated using methods such as crisscross slats (Figure S5C)^59^ and controlled colloidal crystal nucleation.^60^ Taken together, these advances point toward future microswimming robots built entirely from nanometer-addressable nucleic acid microparticles.

## Conclusions and Future Perspectives

In this Account, we highlighted our recent progress in the experimental realization and modeling of colloidal microswimmers made with compliant DNA linkages. To make microswimmer systems more applicable for in vivo applications, our team developed improved fabrication, modeling, and experimental techniques, directly addressing several challenges presented in the literature for soft, small-scale microrobots. Our hybrid fabrication method combines templated assembly with structural DNA nanotechnology to address the manufacturing limitations of flexibly linked microswimmers. Our microswimmers exhibit enhanced structural complexity and controlled particle placement, spacing, and size while maintaining the compliance of their DNA linkage, facilitating low Reynolds locomotion and future swarm studies. Our milliswimmer platform addresses limitations associated with population-level structural heterogeneity, allowing us to experimentally assess hydrodynamic microswimmer models and explore relationships between design features and swimming velocity for low Reynolds number swimmers. Finally, we explore how DNA can enhance the responsiveness and physical intelligence of microswimmer systems by demonstrating the autonomous response of a microswimmer to an enzymatic signal. Taken together, these studies bring us closer to the experimental realization of complex, flexible microswimmers that can autonomously execute planned tasks in vivo.

Despite these recent achievements, there are still challenges that must be overcome for this technology to become practical for manufacturing and healthcare applications. Advancing our understanding of microswimmer dynamics will require the identification of key metrics for dynamic scaling along with the development of the complementary experimental, computational, and modeling tools needed to accurately capture the behavior of these systems. Additionally, enabling in vivo tasks such as targeted drug delivery and real-time diagnostics will require monodisperse microswimmer systems that can survive in and respond to various physiological environments. Achieving optimal performance for biohybrid micro- and nanorobots while maintaining strict biocompatibility standards adds an additional layer of complexity to this challenge.

Our parallel work in DNA nanotechnology, XNAs, and living cells holds the answer to addressing these challenges. While our microswimmers exhibit improved structural precision, their locomotion remains polydisperse, which we suspect results from variations in individual microswimmer magnetization and DNA linkage mechanics. In future studies, more controlled nanostructures, such as DNA origami, and more controlled binding of DNA nanostructures can be used to create microswimmers with mechanically uniform linkages while maintaining responsiveness and reconfigurability to a range of mechanical or chemical signals. Additionally, nondestructive DNA-based mechanisms, such as strand displacement, triplex formation, hairpin opening, and molecular logic gates, can enable controlled reconfiguration and biosensing within microswimmer systems.^19^ Integrating nucleic acid mimics, such as PNA, LNA, and XNA, as materials in microswimmer designs can make these systems more resilient to physiological environments and enzyme degradation while maintaining their biocompatibility. Additionally, advances in Gigadaltan-scale DNA origami assembly can be leveraged to make DNA swimmers on the micrometer scale, expanding the environments and scales in which these robots can operate. Finally, by replacing synthetic components, such as polymer microspheres, with biological materials, our research can enable the creation of fully organic, truly biocompatible microswimmers with enhanced robustness, cargo-carrying capabilities, and tunable sensitivity to mechanical stimuli.

In the near future, we envision combining these advances in nucleic acid nanotechnology and microrobotics to optimize the design, materials, and protective measures of biohybrid micro/nanorobots. In doing so, researchers can enhance the stability, reliability, and performance of these systems while creating a platform with which specialized, physically intelligent microswimmer systems can be made for targeted applications, thus advancing the field of microrobotics for innovations in healthcare, synthetic biology, and biomanufacturing.

## Supplementary Material



## Notes

**Supporting Section S1:** Discussion of relevant DNA microswimmers and magnetically actuated microsystems. **Supporting Section S2:** Detailed description of the SST DNA nanotubes. **Supporting Figure S1:** Structure of the 10-helix SST DNA nanotubes. **Supporting Figure S2:** Examples of colloidal microstructures assembled using optical and plasmonic tweezers, electric and magnetic fields, and physical templates. **Supporting Figure S3:** Free body diagrams for first- and second-order models. **Supporting Figure S4:** Common strategies for interfacing DNA nanotechnologies with living cells. **Supporting Figure S5:** Examples of fixed-size nucleic acid structures fabricated at the micron scale. (PDF)
