# Supplementary material for: Microswimmers That Flex: Advancing Microswimmers with Templated Assembly and Responsive DNA Nanostructures
Source: Acc Mater Res. 2025 Jul 14;6(8):927–38. doi: 10.1021/accountsmr.5c00009 (PMC12379156; doi:10.1021/accountsmr.5c00009)
Supplement: Supplementary file 1 [file mr5c00009_si_001.pdf]

Supporting Information for Microswimmers That Flex: Advancing Microswimmers with Templated Assembly and Responsive DNA Nanostructures

Taryn Imamura, Sarah Bergbreiter, Rebecca E. Taylor*

E-mail: bex@andrew.cmu.edu

Table of Contents

Supporting Section S1: Discussion of relevant DNA microswimmers and magnetically actuated microsystems.

Supporting Section S2: Detailed description of the SST DNA nanotubes.

Supporting Figure S1: Structure of the 10-helix SST DNA nanotubes that formed the flexible linkage of the colloidal microswimmers in Harmatz et al. 2020, Imamura et al. 2024, and Imamura et al. 2025.[1–3]

Supporting Figure S2: Examples of colloidal microstructures assembled using optical and plasmonic tweezers, electric and magnetic fields, and physical templates.

Supporting Figure S3: Free body diagrams used to derive the quasistatic and inertial models in Benjaminson et al. 2023.[4]

Supporting Figure S4: Common strategies for interfacing DNA nanotechnologies with living cells.

Supporting Figure S5: Examples of fixed-size nucleic acid structures fabricated at the micron scale.

Discussion of Relevant DNA and Magnetic Microswimmers

In this section, we present a brief comparison of our microswimmers with other relevant DNA-based and magnetically actuated microswimmers. We also discuss how these systems measure against the key criteria required for future in vivo applications that we present in our Account.

As discussed in the main text of this Account, microswimmers intended for biomedical use must meet several essential requirements to ensure safe and practical deployment in the human body. In particular, they must be small, flexible, and capable of controlled locomotion to navigate through confined and delicate environments. Their fabrication methods must be capable of reliably constructing microswimmer populations with well-defined complex geometries. Additionally, microswimmers must be multifunctional and capable of performing tasks such as morphology change in response to environmental signals.

Unlike the DNA-based microswimmers presented in this Account, several notable examples of small-scale microswimmers ($<20\,\mu m$) in the literature do not use DNA in their construction and meet some, but not all, of the key criteria for safe and practical deployment in the human body. For example, Cheang et al. describe microswimmers composed of chains of magnetic nanoparticles aligned via external magnetic fields.[5] Although these microswimmers are small, their body geometries are poorly defined, with significant structural variability across populations. The lack of physical linkers, such as DNA, limits their mechanical integrity and precludes the incorporation of built-in mechanisms for environmental sensing or programmable response. While they exhibit controlled locomotion under rotating magnetic fields, their motion is more susceptible to Brownian effects, resulting in less stable trajectories. Reconfiguration in response to changing magnetic fields is theoretically possible, although it was explicitly demonstrated. These microswimmers are promising for basic actuation, but they fall short in modularity, robustness, and multifunctionality.

Du et al. present another compelling example of flexible, magnetically actuated microswimmers assembled without physical linkers.[6] These microswimmers demonstrate controlled locomotion, adaptive morphology changes, and dynamic responses to magnetic field inputs. However, field-directed assembly produces swimmers with variable morphologies and inconsistent numbers of component spheres, limiting structural reproducibility. Additionally, their responsiveness is confined to magnetic stimuli, narrowing their potential biological or biochemical response.

Cheang et al. present achiral microswimmers composed of three magnetic microspheres physically linked via biotin-streptavidin binding.[7] While magnetic self-assembly leads to structural variability, relatively homogeneous subpopulations of three-bead microswimmers can still be isolated. These microswimmers do not exhibit reconfiguration or responsiveness beyond magnetic actuation. However, the authors successfully demonstrate multi-robot manipulation and exploit intrinsic magnetic variations to achieve differential control, allowing them to selectively move microswimmers in the same or opposite directions based on the rotating magnetic field input.

While many microswimmers lack precise geometry control, Ni et al. demonstrated a notable exception by using sequential capillarity-assisted particle assembly (sCAPA) to fabricate microswimmers with well-defined 2D geometries.[8] This templated assembly method utilizes capillary forces to position particles, which are then permanently linked via sintering, allowing for the consistent construction of large populations of microswimmers with homogeneous, defined body geometries. Actuated by AC electric fields in a specialized test environment, these microswimmers exhibit diverse trajectories and can manipulate surrounding particles. However, electric field actuation limits their applicability in biological environments, and sintered linkages reduce reconfigurability and adaptability.

Alvarez et al. and van Kesteren et al. extended the sCAPA approach to create reconfigurable, self-propelled microswimmers composed of polystyrene microparticles and thermoresponsive microgels.[9,10] These microswimmers exhibit controlled geometries and are actu-

ated by AC electric fields, similar to Ni et al.[8] Their soft components reversibly swell or shrink in response to thermal cues, enabling morphological changes and programmable locomotion. However, reliance on thermal signals may limit their biomedical applicability, highlighting the need for microswimmers responsive to a broader range of stimuli.

Several compelling examples also exist within the DNA microswimmer subfield. Dreyfus et al. developed microswimmers composed of magnetic microspheres linked by double-stranded DNA (dsDNA) via biotin-streptavidin bonds.[11] While the microspheres were aligned using magnetic fields, compliant ds-DNA strands held the chain in the desired configuration. While this method produces microswimmers with limited morphological complexity, it allows for controlled assembly and retains structural flexibility. The DNA linkage also enables biohybrid integration with red blood cells, allowing for targeted cargo transport. Controlled locomotion was demonstrated under oscillating magnetic fields. Although active reconfiguration was not shown, the use of DNA offers a promising path for responsiveness to biochemical signals and programmability.

Maier et al. demonstrated how the physical properties of DNA nanostructures can be utilized to program the behavior of microswimmers.[12] Their microswimmers consisted of magnetic beads functionalized with single-stranded tile (SST) DNA nanotubes of varying lengths and chiralities, attached via biotin-streptavidin bonds. Under high-frequency rotating magnetic fields, the nanotubes formed flagella-like bundles, enabling propulsion. Although these microswimmers exhibited impressive motility, their velocities were highly variable due to uncontrolled bundle formation, and their geometric complexity was limited. Nevertheless, the study showed that nanotube chirality influenced swimming speed and that flagellar flexibility could be modeled to predict performance. Although reconfiguration and environmental responsiveness were not demonstrated, this work highlights how tuning the properties of DNA subcomponents can be a powerful tool for programming microswimmer behavior.

Across several publications, Tierno et al. present magnetically actuated microswimmers composed of colloidal particles connected by strands of dsDNA.[13,14] They modeled and

experimentally demonstrated that anisotropic dimers of paramagnetic particles can achieve controlled propulsion when suspended near a surface and subjected to a precessing magnetic field. While these microswimmers exhibited controlled locomotion and trajectory following, their assembly via magnetic alignment resulted in inconsistent structures, and their reliance on surface interactions limited the environments in which they could be deployed. Although reconfiguration and programmability were not directly shown, the use of DNA introduces potential for mechanisms such as enzyme-triggered disassembly.

Like Maier et al., Pauer et al. created microswimmers by functionalizing magnetic microspheres with six-helix bundle DNA origami structures.[15] Using PDMS templates, they selectively coated one hemisphere of each particle, offering greater structural control despite limited morphological complexity. Under oscillating magnetic fields, the microswimmers exhibited tunable net propulsion, with velocity modulated by the field frequency and the DNA-coated surface area.

Finally, Lauback et al. designed DNA origami microlevers, microrotors, and microhinge systems that integrate magnetic micro- and nanoparticles to achieve continuous, magnetically controlled rotational motion.[16] Because these structures were adhered to a surface, they do not exhibit locomotion and are not explicitly microswimmers. However, they do demonstrate precise, real-time control over angular position. The use of DNA origami enables the consistent fabrication of nanostructures and offers a promising platform for embedding programmability and computation into microscale systems.

Single-Stranded Tile (SST) DNA Nanotube Structure

The microswimmers presented in Harmatz et al. and Imamura et al. were connected by a collection of single-stranded tile (SST) DNA nanotubes.[1,2] The nanotube design was adapted from Yin et al.[17] Each nanotube is composed of 10 interconnected DNA helices and is approximately 10 nm in diameter. The nanotubes are composed of repeating segments of 40 oligomer strands, each 55 base pairs long. Because the SST nanotubes are made of repeating segments of DNA, they continue growing as long as the necessary strands are present and can grow to be multiple microns (3-5 µm) in length. Each nanotube is biotinylated with single strands of DNA that have biotin attached to their 5' ends. These strands connect to the ends of the nanotubes, allowing them to interface with the streptavidin-coated microspheres.

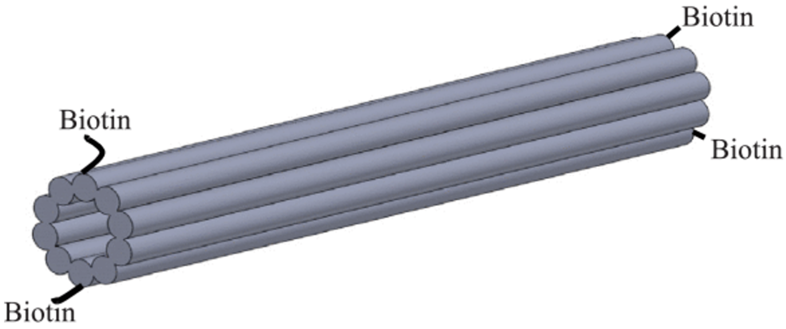

Figure S1: Schematic of the single-stranded tile (SST) DNA nanotubes with biotin attachments that formed the flexible linkage of the microswimmer. Adapted with permission from ref.[1] Copyright 2020 The Authors.

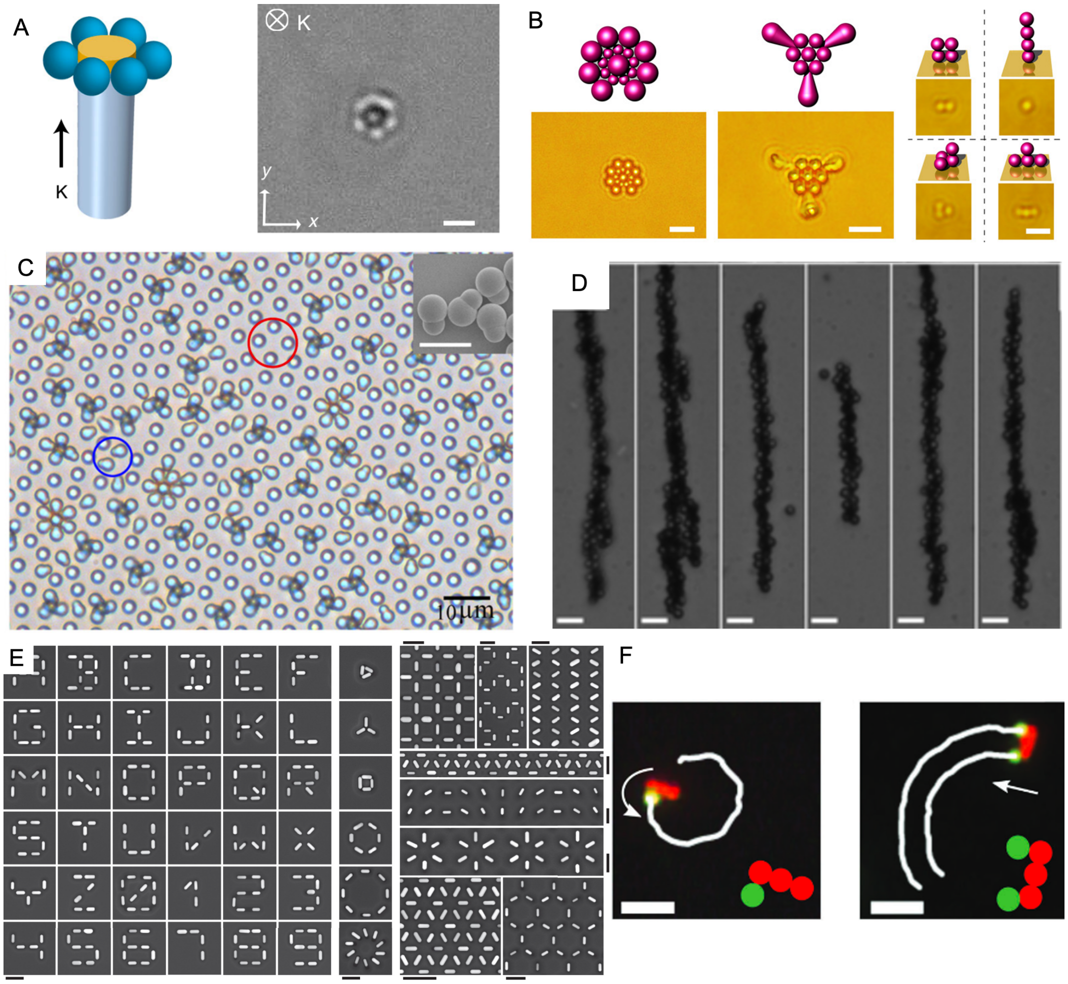

Figure S2: Examples of common methods for assembling colloidal structures. (A) Assembly of gold nanodiscs and 400 nm polystyrene particles into predetermined arrangements through a hybrid optical and plasmonic tweezer approach. Scale bar is 1 µm. Reproduced with permission from ref.[18] Copyright 2019 The Authors. (B) Opto-thermophoretic assembly of polystyrene beads and anisotropic particles in 2D (left and middle) and 3D (right). Scale bars are 5 µm. Reproduced with permission from ref.[19] Copyright 2017 The Authors. (C) Electric-field induced assembly of chiral colloidal clusters. The inset shows an SEM image of the asymmetric dimer building blocks. Scale bar is 10 µm. Reproduced with permission from ref.[20] Copyright 2015 The Authors. (D) Magnetic field assembly of single and multipatch patchy particles. Patches are formed using the template-assisted glancing angle deposition method (GLAD). Scale bars are 1 µm. Reproduced with permission from ref.[21] Copyright 2013 American Chemical Society. (E) SEM micrographs showing the capillary assembly of gold nanorods deposited into a variety of 2D configurations. All scale bars are 250 nm. Reproduced with permission from ref.[22] Copyright 2017 Macmillan Publishers Limited, part of Springer Nature. (F) Fluorescence microscopy snapshots of colloidal microswimmers assembled with sequential capillarity-assisted particle assembly (sCAPA). White overlays show microswimmer trajectories, and the arrows represent the directions of motion. Scale bars are 5 µm Reproduced with permission from ref.[8] Copyright 2017 The Royal Society of Chemistry.

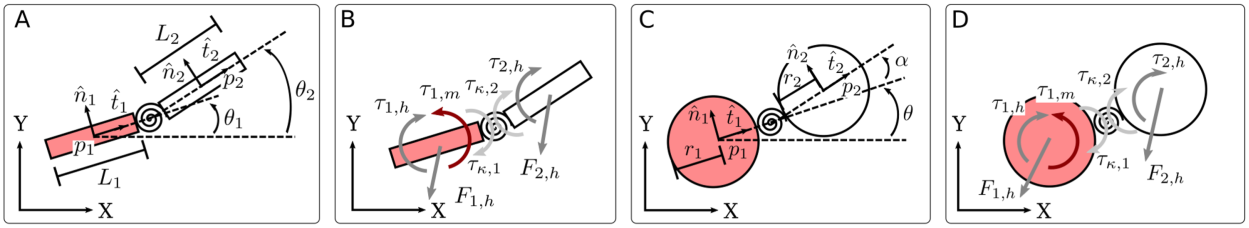

Figure S3: Free body diagrams used to derive first- and second-order milliswimmer models. (A) Coordinate system for the cylindrical swimmer. Each body frame is fixed at the center of mass of its corresponding link and defined by the transverse ($\mathbf{\hat{t}_i}$) and normal ($\mathbf{\hat{n}_i}$) unit vectors of the i-th link. Hydrodynamic drag is anisotropic, with drag in the normal direction being twice that in the transverse direction (this parameter is fit to experimental data in the second-order model). (B) Free-body diagram of the two-link cylindrical swimmer, illustrating the applied forces and torques under the assumption that both links are translating and rotating in the positive directions. The system experiences hydrodynamic forces ($\mathbf{F_{i,h}}$) and torques ($\tau_{\mathbf{i,h}}$), a magnetic torque due to misalignment ($\tau_{\mathbf{1,m}}$), and elastic torques from the torsional spring connecting the links ($\tau_{\kappa,\mathbf{i}}$), where i indicates the link on which the force or torque acts. (C) Coordinate system for the spherical swimmer. Each body frame is fixed at the center of mass of its corresponding link. In contrast to the cylindrical case, hydrodynamic drag is isotropic across all directions. (D) Free-body diagram of the two-link spherical swimmer, showing the forces and torques acting on each link. Figure reproduced with permission from ref.[4] Copyright 2023 The Royal Society of Chemistry.

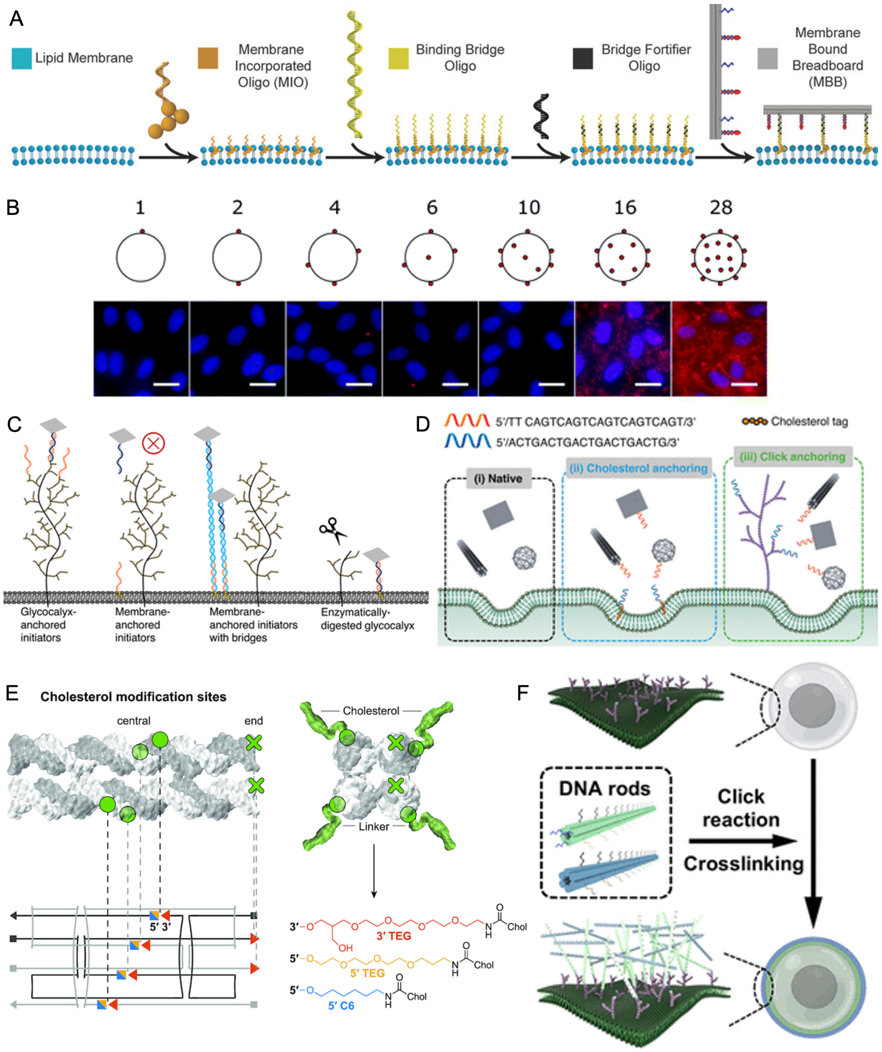

Figure S4: Examples of cell-interfacing DNA nanotechnologies. (A) The sequential functionalization steps to embed DNA-origami nanodevices onto the cell surface. Reproduced with permission from ref.[23] Copyright 2017 WILEY-VCH Verlag GmbH and Co. (B) Fluorescence microscope images show how increasing the number of binding overhangs on DNA origami nanospheres impacts their binding to cells via two-step targeting with cholesterol anchors. Reproduced with permission from ref.[24] Copyright 2021 The Royal Society of Chemistry. (C) Schematic shows direct glycocalyx anchoring of DNA origami nanotiles to the phospholipid bilayer using cholesterol. Reproduced with permission from ref.[25] Copyright 2021 The Authors. (D) Schematic showing three cell surface binding strategies for DNA nanostructures: (i) native adhesion for unmodified DNA nanostructures without overhangs, (ii) two-step membrane targeting with cholesterol anchoring, and (iii) two-step click glycocalyx anchoring. Reproduced with permission from ref.[26] Copyright 2024 The Authors. (E) The side (left) and top view (right) of a DNA nanostructure with cholesterol modification sites are shown in green. Sites are located at the center and end of the construction. Reproduced with permission from ref.[27] Copyright 2019 The Authors. (F) DNA origami nanoshell made from two layers of crosslinked DNA nanorods. The nanoshell acts as armor to protect cells from mechanical stress. Reproduced with permission from ref.[28] Copyright 2023 The Authors.

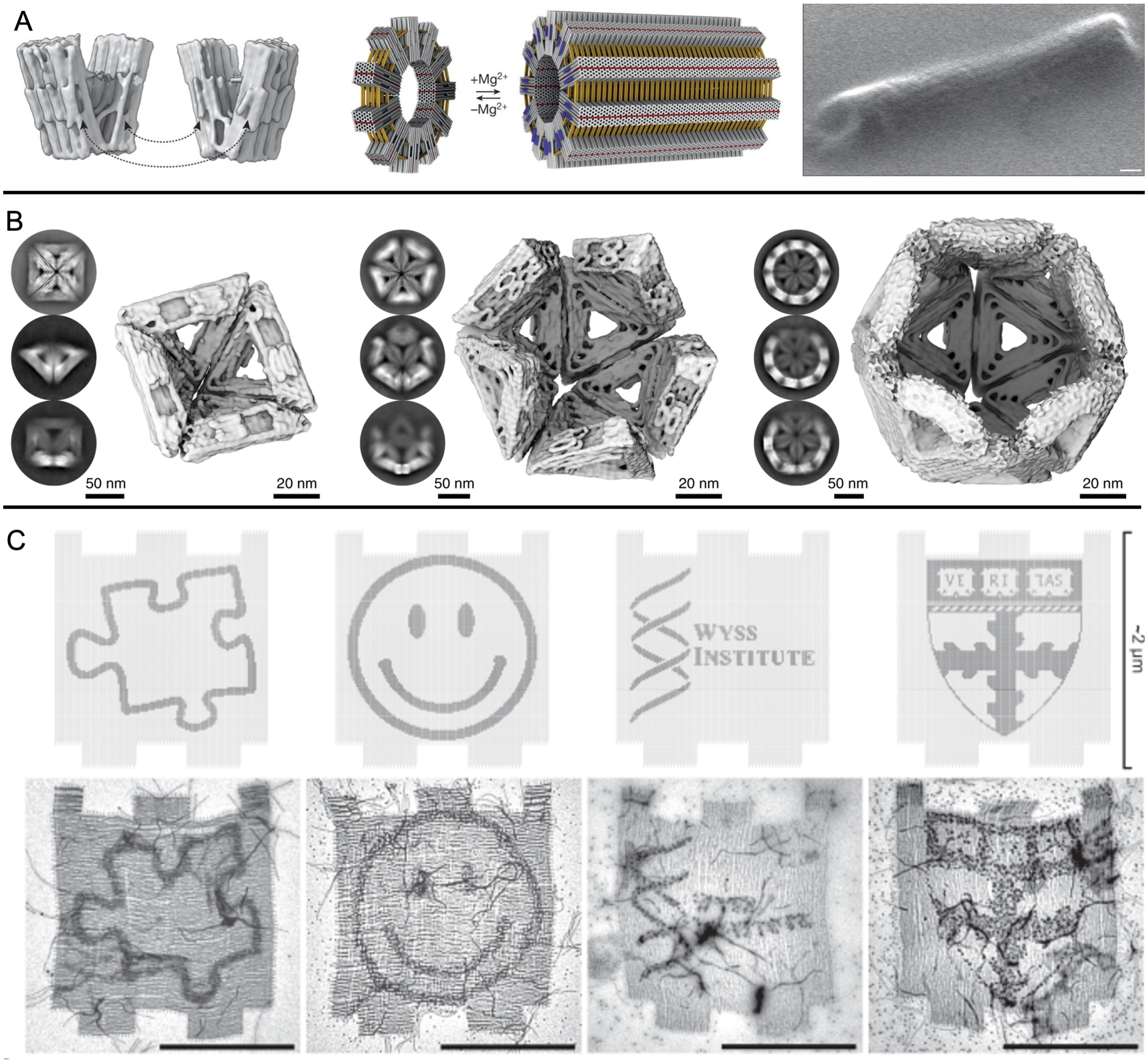

Figure S5: Examples of fixed-size nucleic acid structures fabricated at the micron scale. (A) A cryo-EM density map of a DNA V-brick (left). Hierarchical assembly of these bricks into Gigadalton-scale rings, which can then form micron-scale tubes (middle). Helium-ion microscopy micrographs of the oligomerized tubes (right). Scale bars are 50 nm. Reproduced with permission from ref.[29] Copyright 2017 Macmillan Publishers Limited, part of Springer Nature. (B) Cryo-EM 3D reconstructions of programmable icosahedral shells whose internal cavities exhibited virus-trapping capabilities. Reproduced with permission from ref.[30] Copyright 2021 The Authors. (C) Designs of patterned sheets were produced using the crisscross slats method, where darker dots denote sites that were programmed with a handle sequence used to bind to contrasting DNA nanocubes (top) and TEM images of the assembled sheets (bottom). Scale bars are 1 µm. Reproduced with permission from ref.[31] Copyright 2022 The Authors.

References

(1) Harmatz, M.; Travers, M.; Taylor, R. E. A Customizable DNA and Microsphere-Based, Magnetically Actuated Microswimmer. *J. Microelectromech. Syst.* **2020**, *29*, 990–995, DOI: 10.1109/JMEMS.2020.3011610.

(2) Imamura, T.; Chung, N.; Sonmez, U. M.; Travers, M.; Bergbreiter, S.; Taylor, R. E. Complex Assemblies of Colloidal Microparticles with Compliant DNA Linkers and Magnetic Actuation. *Adv. Mater. Technol.* **2024**, *Early View*, 2401584, DOI: 10.1002/admt.202401584, (accessed 2025-01-04).

(3) Imamura, T.; Kent, T. A.; Taylor, R. E.; Bergbreiter, S. Measuring DNA Microswimmer Locomotion in Complex Flow Environments. 2025 IEEE International Conference on Robotics and Automation (ICRA). Atlanta, GA, 2025.

(4) Benjaminson, E.; Imamura, T.; Lorenz, A.; Bergbreiter, S.; Travers, M.; Taylor, R. E. Buoyant magnetic milliswimmers reveal design rules for optimizing microswimmer performance. *Nanoscale* **2023**, *15*, 14175–14188, DOI: 10.1039/D3NR02846A.

(5) Cheang, U. K.; Kim, M. J. Self-assembly of robotic micro- and nanoswimmers using magnetic nanoparticles. *J Nanopart Res* **2015**, *17*, 145, DOI: 10.1007/s11051-014-2737-z.

(6) Du, D.; Hilou, E.; Biswal, S. L. Reconfigurable paramagnetic microswimmers: Brownian motion affects non-reciprocal actuation. *Soft Matter* **2018**, *14*, 3463–3470, DOI: 10.1039/C8SM00069G.

(7) Kei Cheang, U.; Lee, K.; Julius, A. A.; Kim, M. J. Multiple-robot drug delivery strategy through coordinated teams of microswimmers. *Applied Physics Letters* **2014**, *105*, 83705, DOI: 10.1063/1.4893695.

(8) Ni, S.; Marini, E.; Buttinoni, I.; Wolf, H.; Isa, L. Hybrid colloidal microswimmers through sequential capillary assembly. *Soft Matter* **2017**, *13*, 4252–4259, DOI: 10.1039/C7SM00443E.

(9) Alvarez, L.; Fernandez-Rodriguez, M. A.; Alegria, A.; Arrese-Igor, S.; Zhao, K.; Kröger, M.; Isa, L. Reconfigurable artificial microswimmers with internal feedback. *Nat. Commun* **2021**, *12*, 4762, DOI: 10.1038/s41467-021-25108-2.

(10) van Kesteren, S.; Alvarez, L.; Arrese-Igor, S.; Alegria, A.; Isa, L. Self-propelling colloids with finite state dynamics. *Proceedings of the National Academy of Sciences* **2023**, *120*, e2213481120, DOI: 10.1073/pnas.2213481120.

(11) Dreyfus, R.; Baudry, J.; Roper, M. L.; Fermigier, M.; Stone, H. A.; Bibette, J. Microscopic artificial swimmers. *Nature* **2005**, *437*, 862–865, DOI: 10.1038/nature04090.

(12) Maier, A. M.; Weig, C.; Oswald, P.; Frey, E.; Fischer, P.; Liedl, T. Magnetic Propulsion of Microswimmers with DNA-Based Flagellar Bundles. *Nano Lett.* **2016**, *16*, 906–910, DOI: 10.1021/acs.nanolett.5b03716.

(13) Tierno, P.; Golestanian, R.; Pagonabarraga, I.; Sagués, F. Controlled Swimming in Confined Fluids of Magnetically Actuated Colloidal Rotors. *Phys. Rev. Lett.* **2008**, *101*, 218304, DOI: 10.1103/PhysRevLett.101.218304.

(14) Tierno, P.; Golestanian, R.; Pagonabarraga, I.; Sagués, F. Magnetically Actuated Colloidal Microswimmers. *J. Phys. Chem. B* **2008**, *112*, 16525–16528, DOI: 10.1021/jp808354n.

(15) Pauer, C.; Venczel, A.; Dass, M.; Liedl, T.; Tavacoli, J. Propulsion of Magnetic Beads Asymmetrically Covered with DNA Origami Appendages. *Adv. Mater. Technol.* **2022**, *7*, 2200450, DOI: 10.1002/admt.202200450.

(16) Lauback, S.; Mattioli, K. R.; Marras, A. E.; Armstrong, M.; Rudibaugh, T. P.; Sooryakumar, R.; Castro, C. E. Real-time magnetic actuation of DNA nanodevices via modular integration with stiff micro-levers. *Nature Communications* **2018**, *9*, DOI: 10.1038/s41467-018-03601-5, Publisher: Nature Publishing Group.

(17) Yin, P.; Hariadi, R. F.; Sahu, S.; Choi, H. M. T.; Park, S. H.; LaBean, T. H.; Reif, J. H. Programming DNA Tube Circumferences. *Science* **2008**, *321*, 824–826, DOI: 10.1126/science.1157312.

(18) Ghosh, S.; Ghosh, A. All optical dynamic nanomanipulation with active colloidal tweezers. *Nat Commun* **2019**, *10*, 4191, DOI: 10.1038/s41467-019-12217-2.

(19) Lin, L.; Zhang, J.; Peng, X.; Wu, Z.; Coughlan, A. C. H.; Mao, Z.; Bevan, M. A.; Zheng, Y. Opto-thermophoretic assembly of colloidal matter. *Sci. Adv.* **2017**, *3*, e1700458, DOI: 10.1126/sciadv.1700458.

(20) Ma, F.; Wang, S.; Wu, D. T.; Wu, N. Electric-field–induced assembly and propulsion of chiral colloidal clusters. *Proc. Natl. Acad. Sci. U. S. A.* **2015**, *112*, 6307–6312, DOI: 10.1073/pnas.1502141112.

(21) He, Z.; Kretzschmar, I. Template-Assisted GLAD: Approach to Single and Multipatch Patchy Particles with Controlled Patch Shape. *Langmuir* **2013**, *29*, 15755–15761, DOI: 10.1021/la404592z.

(22) Flauraud, V.; Mastrangeli, M.; Bernasconi, G. D.; Butet, J.; Alexander, D. T. L.; Shahrabi, E.; Martin, O. J. F.; Brugger, J. Nanoscale topographical control of capillary assembly of nanoparticles. *Nat. Nanotechnol.* **2017**, *12*, 73–80, DOI: 10.1038/nnano.2016.179.

(23) Akbari, E.; Mollica, M. Y.; Lucas, C. R.; Bushman, S. M.; Patton, R. A.; Shahhosseini, M.; Song, J. W.; Castro, C. E. Engineering Cell Surface Function with DNA Origami. *Adv. Mater.* **2017**, *29*, 1703632, DOI: 10.1002/adma.201703632.

(24) Liu, Y.; Wijesekara, P.; Kumar, S.; Wang, W.; Ren, X.; Taylor, R. E. The effects of overhang placement and multivalency on cell labeling by DNA origami. *Nanoscale* **2021**, *13*, 6819–6828, DOI: 10.1039/D0NR09212F.

(25) Wijesekara, P.; Liu, Y.; Wang, W.; Johnston, E. K.; Sullivan, M. L. G.; Taylor, R. E.; Ren, X. Accessing and Assessing the Cell-Surface Glycocalyx Using DNA Origami. *Nano Lett.* **2021**, *21*, 4765–4773, DOI: 10.1021/acs.nanolett.1c01236.

(26) Wang, W.; Chopra, B.; Walawalkar, V.; Liang, Z.; Adams, R.; Deserno, M.; Ren, X.; Taylor, R. E. Cell–Surface Binding of DNA Nanostructures for Enhanced Intracellular and Intranuclear Delivery. *ACS Appl. Mater. Interfaces* **2024**, *16*, 15783–15797, DOI: 10.1021/acsami.3c18068.

(27) Ohmann, A.; Göpfrich, K.; Joshi, H.; Thompson, R. F.; Sobota, D.; Ranson, N. A.; Aksimentiev, A.; Keyser, U. F. Controlling aggregation of cholesterol-modified DNA nanostructures. *Nucleic Acids Res.* **2019**, *47*, 11441–11451, DOI: 10.1093/nar/gkz914.

(28) Wang, W.; Hayes, P. R.; Ren, X.; Taylor, R. E. Synthetic Cell Armor Made of DNA Origami. *Nano Lett.* **2023**, *23*, 7076–7085, DOI: 10.1021/acs.nanolett.3c01878.

(29) Wagenbauer, K. F.; Sigl, C.; Dietz, H. Gigadalton-scale shape-programmable DNA assemblies. *Nature* **2017**, *552*, 78–83, DOI: 10.1038/nature24651.

(30) Sigl, C.; Willner, E. M.; Engelen, W.; Kretzmann, J. A.; Sachenbacher, K.; Liedl, A.; Kolbe, F.; Wilsch, F.; Aghvami, S. A.; Protzer, U.; Hagan, M. F.; Fraden, S.; Dietz, H. Programmable icosahedral shell system for virus trapping. *Nat. Mater.* **2021**, *20*, 1281–1289, DOI: 10.1038/s41563-021-01020-4.

(31) Wintersinger, C. M.; Minev, D.; Ershova, A.; Sasaki, H. M.; Gowri, G.; Berengut, J. F.; Corea-Dilbert, F. E.; Yin, P.; Shih, W. M. Multi-micron crisscross structures grown from DNA-origami slats. *Nat. Nanotechnol.* **2023**, *18*, 281–289, DOI: 10.1038/s41565-022-01283-1.
